# Sustained remission of multi-line relapsed extranodal NK/T-cell lymphoma, nasal type, following sintilimab and chidamide: A case report

**DOI:** 10.1097/MD.0000000000024824

**Published:** 2021-03-12

**Authors:** Jingyan Xu, Xihui Xu, Jieyu Chen, Jing Wang, Chong Jiang, Chenglan Lv, Bing Chen

**Affiliations:** a Department of Hematology; b Department of Pathology; c Department of Radiology, Nanjing Drum Tower Hospital, The Affiliated Hospital of Nanjing University Medical School, Nanjing, Jiangsu Province, People's Republic of China.

**Keywords:** case report, extranodal NK/T-cell lymphoma nasal type, histone deacetylase inhibitor, programmed cell death protein 1

## Abstract

**Introduction:**

There is currently no optimal treatment modality for refractory or relapsed Extranodal NK/T-cell lymphoma, nasal type (ENKTL). In recent years, programmed cell death protein 1 (PD-1)/programmed cell – ligand 1 pathway blockade and histone deacetylase inhibitors have emerged as promising strategies for refractory or relapsed ENKTL. Accumulating evidence has shown that therapeutic effects of anti-PD-1 antibody could be enhanced by histone deacetylase inhibitors.

**Patient Concerns:**

A 52-year-old male patient was diagnosed with stage I ENKTL by biopsy on February 2010.

**Diagnosis:**

positron emission tomography–computed tomography (PET-CT) and biopsy were used to diagnose relapsed ENKTL in 2014.

**Interventions:**

The patient was treated with radiotherapy and six cycles of etoposide, prednisone, vincristine (Oncovin), cyclophosphamide and doxorubicin hydrochloride and achieved complete remission (CR) by PET-CT in August 2010. In November 2014, the patient was diagnosed with relapsed stage IV ENKTL and was treated with six cycles of alternative chemotherapy with the regimen of steroid (dexamethasone), methotrexate, ifosfamide, L-asparaginase, and etoposide and pegaspargase plus Gemcitabine, Oxaliplatin along with radiotherapy. The patient achieved remission and was placed on thalidomide maintenance treatment. Upon suspicion of relapse suggested by PET-CT, Autologous stem cell transplant was performed after BCNU, etoposide, Ara-C, and melphalan preconditioning on February 2016. Following relapse again in December 2016, the lesions of left femur were treated with radiotherapy and he received anti-PD-1 antibody. He was treated with 4 cycles of pegaspargase plus Gemcitabine, Oxaliplatin on August 2017. The patient's condition improved. He received maintenance and consolidation therapy including lenalidomide, radiotherapy of the right nasal cavity and paranasal sinuses and antigen-specific reactive T cell infusions. PET-CT imaging showed there was high metabolic activity signal in the distal end of right femoral on August 2018 and the treatment regimen was adjusted to radiotherapy of the distal end of right femoral and systemic treatment of PD-1 antibody Sintilimab and chidamide 30 mg. After 5 months post-treatment, biopsy of nasopharynx showed no lymphoma cells. The patient continued the treatment of Sintilimab and chidamide 20 mg.

**Outcomes:**

PET-CT imaging showed his lesions obtained remission after 8 months post-treatment.

**Conclusion:**

Thus, combination of sintilimab and chidamide can be used to treat relapsed ENKTL following treatment failure from chemo-, radio-, and immuno-therapy. A clinical trial has been launched.

## 1 Introduction

Extranodal NK/T-cell lymphoma, nasal type (ENKTL) is a subtype of mature T-cell and NK-cell lymphoma that is more common in Asia and South American populations. In China, ENKTL is the most common subtype of T cell-derived non-hodgkin lymphoma (T-NHL) and accounts for 28.16% T-NHL.^[1]^ ENKTL is an aggressive lymphoma and its prognosis is relatively poor.^[2]^ For ENKTL patients failing L-asparaginase-based regimen, there are no known effective salvage approaches. The median overall survival for refractory or relapsed ENKTL is only half a year.^[3]^ In the past few years, anti-programmed death (PD-1) antibodies were applied to treat refractory or relapsed ENKTL and demonstrated promising results. For example, 28 refractory or relapsed ENKTL were treated by PD-1 antibody sintilimab in ORIENT-4 trial.^[4]^ The objective response rate and complete response rate were 67.9% and 14.3%, respectively. Remarkably, 2-year overall survival rate was 78.6%. Furthermore, sintilimab has been recommended to treat refractory or relapsed ENKTL according to 2020 Chinese Society of Clinical Oncology lymphoma guideline. Chidamide, a novel benzamide type of subtype-selective histone deacetylase inhibitor (HDACi), has been approved by the China Food and Drug Administration as a treatment for refractory or relapsed peripheral T cell lymphoma. There are increasing evidences of synergistic effects of PD-1 antibody and HDACi. For example, HDACi are associated with increased programmed cell – ligand 1 (PD-L1) expression and augmented responses to PD-1 antibody.^[5]^ In the present case, compassionate use of sintilimab plus chidamide was applied to treat a relapsed ENKTL after multi-line treatments including the anti-PD-1 antibody pembrolizumab. To the best of our knowledge, this case is the first example of successfully using sintilimab plus chidamide for the treatment of multi-line relapsed ENKTL.

## 2 Case report

**Figure 1:**
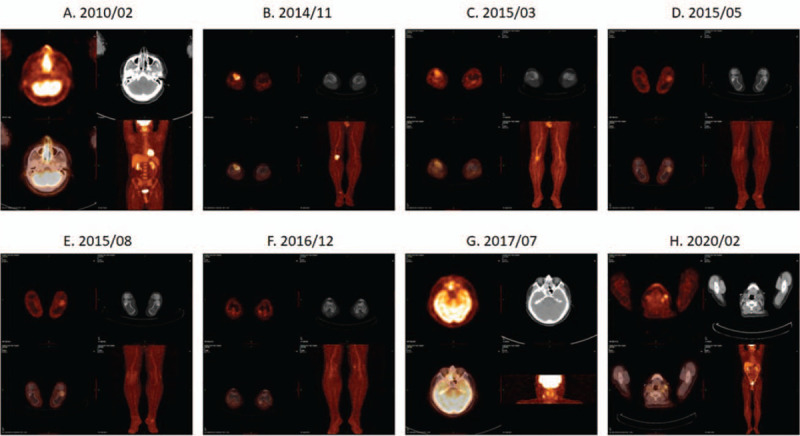
Positron emission tomography (PET) scan assessment. A. Baseline PET-CT showed increased uptakes in the nasopharynx (SUV_max_: 14.5). B. After radiotherapy and six cycles of EPOCH: Complete remission but presence of metabolic activity lesions (SUVmax: 8.9) in upper right tibiae and right calcaneum bone of right leg. C-E. After six cycles of SMILE /P-GEMOX and radiotherapy, he achieved remission. F,G. However, PET-CT imaging showed mild increase of local metabolic activity in the right lateral malleolus, the third and fourth left toe bones and the fifth right toe bone. H. Patient remains in remission as of February 2020 following treatment with Sintilimab and chidamide. PET-CT shows no evidence of relapse or metastasis. SMILE = steroid (dexamethasone), methotrexate, ifosfamide, L-asparaginase, and etoposide, SUV = standard unit value.

A 52-year-old male was diagnosed with stage I ENKTL by biopsy on February 2010 (Table 1). Positron emission tomography–computed tomography 9PET-CT0 imaging showed increased uptakes in the nasopharynx ([standard unit value] SUV_max_: 14.5, Fig. 1A). He was treated with radiotherapy and six cycles of etoposide, prednisone, vincristine (Oncovin), cyclophosphamide and doxorubicin hydrochloride and achieved CR by PET-CT in August 2010. The patient's disease relapsed on November 2014. PET-CT imaging of right leg showed lesions of metabolic activity (SUV_max_: 8.9) in upper right tibiae and right calcaneum bone (Fig. 1B). The biopsy of the right tibiae confirmed relapsed stage IV ENKTL. He was subsequently started on six cycles of alternative chemotherapy with the regimen of steroid (dexamethasone), methotrexate, ifosfamide, L-asparaginase, and etoposide and pegaspargase plus Gemcitabine, Oxaliplatin and radiotherapy of upper right tibiae and right calcaneum bone (56 Gy/20 f, Fig. 1C, D). He achieved remission including negative Epstein-Barr virus and received thalidomide maintenance treatment (200 mg qd). On November 2015, PET-CT imaging of showed mild increase of local metabolic activity in the right lateral malleolus, the third and fourth left toe bones and the fifth right toe bone (Fig. 1E). His disease was suspected relapsed, and there was no evidence of lymphoma infiltration by bone marrow biopsy. His peripheral blood stem cells were mobilized by combination of CTX, VP 16 and G-CSF on December 2015. Autologous stem cell transplant was performed after BCNU, etoposide, Ara-C, and melphalan preconditioning on February 2016. On December 2016, PET-CT imaging showed increase of metabolic activity at the medial malleolus of left femur (SUV_max_: 3.6), nasopharynx and oropharynx (SUV_max_: 6.5), right mastoid antrum, left sphenoid sinus and the left nasal soft tissue (SUV_max_: 6.5) (Fig. 1F). The biopsy of left femur confirmed relapsed ENKTL. There were 5% lymphoma cells in the bone marrow. The lesions of left femur were treated with radiotherapy and he received PD-1 antibody (Keytruda, 200 mg [every 3 weeks] q3w). His condition aggravated after half a year. PET-CT imaging showed density shadow filling of bilateral ethmoid sinuses, bilateral maxillary sinus, and bilateral nasal passages (SUVmax: 10.7), nasopharyngeal soft tissue was thickened (SUVmax: 8.4) (Fig. 1G). He was treated with 4 cycles of pegaspargase plus Gemcitabine, Oxaliplatin on August 2017. PET-CT imaging showed mild to moderate increase of metabolic activity at the right ethmoid sinus, right nasal meatus and nasopharynx (SUV_max_: 2.6), but the metabolic activity was significantly reduced compared with previous results. The patient's condition improved. He received maintenance and consolidation therapy including lenalidomide, radiotherapy of the right nasal cavity and paranasal sinuses (54 Gy/27 f) and antigen-specific reactive T cell infusions. PET-CT imaging showed there was high metabolic activity signal in the distal end of right femoral on August 2018. Hypothesizing that he developed new lesions, the treatment regimen was adjusted to radiotherapy of the distal end of right femoral and systemic treatment of PD-1 antibody (Sintilimab, 200 mg Q3W) and chidamide (30 mg biw). After 5 months post-treatment, biopsy of nasopharynx showed no lymphoma cells. PET-CT imaging showed his lesions obtained remission after 8 months post-treatment. Then he continued the treatment of (Sintilimab, 100 mg q3w) and chidamide (20 mg W2d). PET-CT imaging showed that the nasopharynx SUV was 3.5 and the lymph nodes of left neck, below the jaw and supraclavicular bone area enlarged on February 2020. The largest lymph node size was 2.1×12 cm, SUV was 5.2 (Fig. 1H). No obvious abnormality was found by nasopharyngoscopy. The biopsy of neck lymph node demonstrated that there was no lymphoma infiltration. His disease maintained complete remission by the date of submission. The most adverse events were grade 1 decreased platelet count, grade 1 decreased white blood count and pituitary hypothyroidism.

**Table 1:** Therapy course, complications, and disease response after each treatment.

Diagnosis	Clinical presentation	Pathology	Other examines	Therapy	Response
2010.2 stage I ENKTL	Nasal obstruction No fever Night sweat Emaciation	CD56++, CD3+, CD7+, CD3+, CD2++, CD30+, TIA1++, Perforin+++, Gran B++, CD20-, CD79a-, CD10-, CD21-, Cyclin D1-, Mum+, CK- Ki-67 some area 80%+ EBER+	Blood EBV- No lymphoma cells in the bone marrow	2010 radiotherapy (lymphoma area 56 Gy/31 f, prevention area 48.6 Gy/27 f) plus EPOCH (6 cycles)	Complete response by PET-CT
2014.11 relapsed stage IV ENKTL	Lateral radiotherapy-induced pain of right lower limb	CD56 (++), CD2 (+++), TIA-1 (+++), ki-67 60% (+), CD30 (+), ALK (−), PD-1 (−)	Blood EBV 1.15×10^3^ IU/mL No lymphoma cells in the bone marrow	SMILE/P-GEMOX (6 cycles) plus radiotherapy of upper right tibiae and right calcaneum bone (56 Gy/20 f) 200 mg qd thalidomide maintenance	Response including blood EBV became negative
2015.9 suspicious relapsed stage IV ENKTL	Pain of right knee post-activity	–	Blood EBV- No lymphoma cells in the bone marrow	ASCT	
2016.12 relapsed stage IV ENKTL		Lymphoma cell CD2++, CD3++, CD7 Partial +, CD56 Diffuse +, TIA-1++, CD20-, ki-67 30%+, EBER Few +, Perforin Very Few+, Gran B+	Blood EBV- 5% lymphoma cells in the bone marrow No obvious abnormity by nasopharyngoscope	Radiotherapy of left femur lesions plus 200 mg KEYTRUDA	Progression after half a year
2017.7 relapsed stage IV ENKTL	Nasal obstruction, No fever	CD20(−), CD3(+), CD2(+++), CD56(+++), GranB(+++), TIA(+++), Perforin(−), Ki67(40%+), CD5(−), CD7(+++), MYC (<10%+), P53(<1%+), BCL-2(Diffuse +), PD-1(−), PD-L1(−), EBER(+)	Blood EBV- Suspicious 2% lymphoma cells in the bone marrow	P-GEMOX (4 cycles) Maintenance/Consolidation: thalidomide, radiotherapy of the right nasal cavity and paranasal sinuses (54 Gy/27 f) and antigen-specific reactive T cell infusions	Progression
2018.8 relapsed stage IV ENKTL	——	——	Blood EBV- No obvious abnormity by nasopharyngoscope	Radiotherapy of the distal end of right femoral and systemic treatment of PD-1 antibody (Sintilimab, 200 mg Q3W) and chidamide (30 mg biw)	Response
				Sintilimab (100 mg Q3W) and chidamide (20 mg biw)	Complete response

ASCT = autologous stem cell transplant, EBV = Epstein-Barr virus, ENKTL = extranodal NK-cell lymphoma, nasal type, EPOCH = etoposide, prednisone, vincristine (Oncovin), cyclophosphamide and doxorubicin hydrochloride, PET-CT = positron emission tomography–computed tomography, PD-L1 = programmed cell – ligand , P-GEMOX = pegaspargase plus Gemcitabine, Oxaliplatin, SMILE = steroid (dexamethasone), methotrexate, ifosfamide, L-asparaginase, and etoposide.

## 3 Discussions

ENKTL is an aggressive and heterogenenous entity of T-NHL, strongly associated with Epstein-Barr virus infection. Currently, there are no optimal treatment modality for refractory or relapsed ENKTL. Recently, there has been significant interest in utilizing immune- and cellular therapy to treat ENKTL due to several properties of ENKTL.^[6]^ First, tumor cells in Epstein-Barr virus-associated malignancies express latent membrane proteins that are good candidate targets for cytotoxic T-lymphocytes (CTLs).^[7,8]^ Second, several studies have shown that serum PD-L1 levels are associated with a prognosis of ENKL.^[9–11]^ A study conducted by West China Hospital demonstrated that ENKTL patients expressing more PD-L1 on circulating lymphocytes may have weaker systemic immunity and may therefore be more prone to disease progression.^[12]^ PD-1/PD-L1 pathway blockade has emerged as a promising strategy for refractory or relapsed ENKTL. As early as 2017, Kwong et al. first applied PD-1 antibody to treat seven patients with refractory or relapsed ENKTL failing L-asparaginase and found all patients responded (5 CR and 2 partial remission), but the follow-up period was only six months.^[13]^ Recently, a multi-omics analysis conducted by Shanghai Rui Jin Hospital revealed that ENKTL has 3 molecular subtypes including TSIM, MB and HEA. TSIM subtype overexpress PD-L1/L2 and may be sensitive to PD-1 blockade. HEA subtype is characterized by aberrant histone acetylation and responses to HDACi chidamide. Unfortunately, the molecular subtype of our patient was unclear. Our patient's disease progressed repeatedly. PD-1 antibody Pembrolizumab and radiotherapy was used to treat the patient, but the disease relapsed after only half a year. Interesting, our patient obtained remission after the combination of another PD-1 antibody Sintilimab, HDACi chidamide and radiotherapy. Most importantly, his disease remission sustained and the maintenance time had already surpassed 1 and a half year. The patient has found the treatment to be tolerable with no serious adverse effects.

We presume that the therapeutic effect may mainly due to synergistic effect of Sintilimab and chidamide. Accumulating evidence has shown that integrated immune cycle is essential for effective response to anti-PD-1 therapy, epigenetic modification can restore impairments in the immune cycle by reprogramming tumor microenvironment, increasing the presentation of tumor antigens, and regulating T cell trafficking and reactivation.^[14]^ For example, a recent preclinical study showed that inhibition of HDAC enhances PD-1 antibody efficacy by rendering cancer cells visible for T cell-mediated destruction.^[15]^ In 2020 EHA meeting, a poster demonstrated that Pembrolizumab/HDACi Romidepsin is a safe, effective and promising combination in relapsed/refractory peripheral T-cell lymphoma. In this study, 19 patients with relapsed/refractory peripheral T-cell lymphoma were treated with the combination of Pembrolizumab and Romidepsin, the best complete response rate and the best objective response rate are 42% and 47%, respectively.^[16]^ A clinical trial evaluating PD-1 antibody and HDACi is ongoing in refractory or relapsed ENKTL (NCT03820596).

## 4 Conclusions

In conclusion, we present a case of relapsed ENKTL following treatment failure from chemo-, radio-, and immuno-therapy successfully treated with combination of sintilimab and chidamide. Our approach combining the anti-PD1 antibody with HDACi reveals the potential of this combination to manage of relapsed ENKTL following treatment failure. A clinical trial has been launched to study this treatment regimen.

## Acknowledgments

We would like to thank our other colleagues for their input into the current case.

## Author contributions

All authors were involved in the care of the patient. JX, XX, JW, CL, and BC designed the treatment plan. JX, XX, and JC wrote the manuscript. JC provided the pathology analysis. CJ provided the radiology images and compiled the figure.

**Conceptualization:** Jingyan Xu, Jieyu Chen, Bing Chen.

**Data curation:** Jingyan Xu, Xihui Xu, Jieyu Chen, Jing Wang, Chong Jiang, Bing Chen.

**Formal analysis:** Jingyan Xu, Jieyu Chen, Bing Chen.

**Funding acquisition:** Jingyan Xu, Bing Chen.

**Investigation:** Jingyan Xu, Jing Wang, Bing Chen.

**Methodology:** Jingyan Xu, Jieyu Chen, Bing Chen.

**Project administration:** Jingyan Xu.

**Supervision:** Jingyan Xu, Bing Chen.

**Validation:** Jingyan Xu, Chong Jiang, Bing Chen.

**Visualization:** Jieyu Chen, Chong Jiang, Bing Chen.

**Writing – original draft:** Jingyan Xu, Xihui Xu, Jieyu Chen, Jing Wang, Chong Jiang, Bing Chen.

**Writing – review & editing:** Jingyan Xu, Xihui Xu, Jieyu Chen, Jing Wang, Chong Jiang, Chenglan Lv, Bing Chen.

## References

[1] Li XQ Li GD Gao ZF. Distribution pattern of lymphoma subtypes in China: a nationwide multicenter study of 10 002 cases [J]. J Diagn Concepts Pract 2012;11:111–5.

[2] Lee J Suh C Park YH. Extranodal natural killer T-cell lymphoma, nasal-type: a prognostic model from a retrospective multicenter study. J Clin Oncol 2006;24:612–8. 16380410 10.1200/JCO.2005.04.1384

[3] Lim SH Hong JY Lim ST. Beyond first-line non-anthracycline-based chemotherapy for extranodal NK/T-cell lymphoma: clinical outcome and current perspectives on salvage therapy for patients after first relapse and progression of disease. Ann Oncol 2017;28:2199–205. 28911074 10.1093/annonc/mdx316

[4] Li JY Tao R Fan L. Sintilimab for relapsed/refractory (r/r) extranodal NK/T cell lymphoma (ENKTL): Extended follow-up on the multicenter, single-arm phase II trail (ORIENT-4). Journal of Clinical Oncology 2020;38:8050.

[5] Suraweera A O’Byrne KJ Richard DJ. Combination therapy with histone deacetylase inhibitors (HDACi) for the treatment of cancer: achieving the full therapeutic potential of HDACi. Front Oncol 2018;8:92. 29651407 10.3389/fonc.2018.00092 PMC5884928

[6] Yamaguchi M Suzuki R Oguchi M. Advances in the treatment of extranodal NK/T-cell lymphoma, nasal type. Blood 2018;131:2528–40. 29602763 10.1182/blood-2017-12-791418

[7] Cho SG Kim N Sohn HJ. Long-term outcome of extranodal NK/T cell lymphoma patients treated with postremission therapy using EBV LMP1 and LMP2a-specific CTLs. Mol Ther 2015;23:1401–9. 26017177 10.1038/mt.2015.91 PMC4817864

[8] Bollard CM Gottschalk S Torrano V. Sustained complete responses in patients with lymphoma receiving autologous cytotoxic T lymphocytes targeting Epstein-Barr virus latent membrane proteins. J Clin Oncol 2014;32:798–808. 24344220 10.1200/JCO.2013.51.5304 PMC3940538

[9] Jo J-C Kim M Choi Y. Expression of programmed cell death 1 and programmed cell death ligand 1 in extranodal NK/T-cell lymphoma, nasal type. Ann Hematol 2017;96:25–31. 27696202 10.1007/s00277-016-2818-4

[10] Nagato T Ohkuri T Ohara K. Programmed death-ligand 1 and its soluble form are highly expressed in nasal natural killer/T-cell lymphoma: a potential rationale for immunotherapy. Cancer Immuno, Immunothera 2017;66:877–90. 10.1007/s00262-017-1987-x PMC11028583 28349165

[11] Wang H Wang L Liu W-J. High post-treatment serum levels of soluble programmed cell death ligand 1 predict early relapse and poor prognosis in extranodal NK/T cell lymphoma patients. Oncotarget 2016;7:33035. 27105512 10.18632/oncotarget.8847 PMC5078073

[12] Feng Y Jing C Yu X. Predicting treatment response of patients with extranodal natural killer/T-cell lymphoma based on levels of PD-L1 mRNA and soluble PD-L1 [J]. Hematol Oncol 2020;38:467–77. 32515093 10.1002/hon.2758 PMC7689790

[13] Kwong YK Chan TSY Tan D. PD1 blockade with pembrolizumab is highly effective in relapsed or refractory NK/T-cell lymphoma failing l-asparaginase. Blood 2017;129:2437–42. 28188133 10.1182/blood-2016-12-756841

[14] Chen X Pan X Zhang W. Epigenetic strategies synergize with PD-L1/PD-1 targeted cancer immunotherapies to enhance antitumor responses. Acta Pharm Sin B 2020;10:723–33. 32528824 10.1016/j.apsb.2019.09.006 PMC7276686

[15] Burke B Eden C Perez C. Inhibition of Histone Deacetylase (HDAC) enhances checkpoint blockade efficacy by rendering bladder cancer cells visible for T cell-mediated destruction. Front Oncol 2020;10:699. 32500025 10.3389/fonc.2020.00699 PMC7243798

[16] Becnel M Xu J Feng L. Correlation of PD-L1 expression with response-phase / trial of pembrolziumab and romidepsin in relapsed/refractory peripheral T-cell lymphoma. 2020, EHA.

